# Upregulation of PDGF Mediates Robust Liver Regeneration after Nanosecond Pulsed Electric Field Ablation by Promoting the HGF/c-Met Pathway

**DOI:** 10.1155/2020/3635787

**Published:** 2020-03-14

**Authors:** Junjie Qian, Jianpeng Liu, Liangjie Hong, Haohao Lu, Danjing Guo, Zhen Liu, Lin Zhou, Shengyong Yin, Shusen Zheng

**Affiliations:** ^1^Division of Hepatobiliary and Pancreatic Surgery, Department of Surgery, The First Affiliated Hospital, School of Medicine, Zhejiang University, Hangzhou 310003, China; ^2^NHFPC Key Laboratory of Combined Multi-Organ Transplantation, Hangzhou 310003, China; ^3^Key Laboratory of the Diagnosis and Treatment of Organ Transplantation, CAMS, Hangzhou 310003, China; ^4^Key Laboratory of Organ Transplantation, Hangzhou, Zhejiang Province 310003, China; ^5^Institute of Industrial Ecology and Environment, Zhejiang University, Hangzhou, Zhejiang Province 310007, China; ^6^Collaborative Innovation Center for Diagnosis Treatment of Infectious Diseases, China

## Abstract

Nanosecond pulsed electric field (nsPEF) has emerged as a promising tool for hepatocellular carcinoma ablation recently. However, little is known about how nsPEF affects liver regeneration while being applied to eliminate liver lesions. Besides, the impact of nsPEF ablation on liver function should also be taken into consideration in the process. In this paper, we study the impact of nsPEF ablation on liver function by the measurement of serum levels of AST and ALT as well as liver regeneration and relevant molecular mechanisms in vivo. We found that mouse liver function exhibited a temporary injury without weight loss after ablation. In addition, local hepatic nsPEF ablation promoted significant proliferation of hepatocytes of the whole liver with an increase in HGF level. Moreover, the proliferation of hepatocytes was dramatically inhibited by the inhibitor of c-Met. Of interest, the periablational area is characterized by high level of PDGF and a large amount of activated hepatic stellate cells. Furthermore, neutralizing PDGF was able to significantly inhibit liver regeneration, the increased HGF level, and the accumulation of activated HSCs. Our findings demonstrated that nsPEF not only was a safe ablation approach but also could stimulate the regeneration of the whole liver through the activation of the HGF/c-Met pathway by upregulation of PDGF within the periablational zone.

## 1. Introduction

Radical or partial hepatectomy is the first line of the therapeutic option for liver diseases, especially for benign and malignant liver tumors [1]. Unfortunately, most of malignant liver tumors are secondary to cirrhosis or hepatitis, and patients have increasing risk to suffer liver function failure and even death due to the impaired hepatic compensation posthepatectomy. Thus, developing an alternative approach for the treatment of a liver tumor with few side effects on liver function and more contribution to liver regeneration is crucial to improve the outcome of patients with hepatocellular carcinoma (HCC).

A nanosecond pulsed electric field (nsPEF) is an emerging bioelectrical technique that has potential in the treatment of various malignancies, including melanoma [2], squamous cell carcinoma [3], HCC [4], and pancreatic cancer [5]. nsPEF induces apoptosis [6] or necrosis [7] of tumor cells through ultrashort electric pulses (nanosecond) with high voltage (10 kV/cm range) and rapid rise times (nanosecond). It is able to electropermeate the cellular membrane and influence intracellular organelles and leads to cell destruction [8]. It could lower the risk of local complications, including the damage of bile ducts and vascular vessels caused by thermal effect or chemical toxicity [9], which commonly occurs in the treatment with radiofrequency ablation (RFA) and percutaneous ethanol injection (PEI), respectively.

Recently, some evidences have demonstrated that RFA is capable of inducing liver regeneration [10]. Rozenblum et al. [11] demonstrated that the treatment with RFA on even a small part of a normal liver can activate the hepatocyte growth factor (HGF)/c-Met kinase pathway and promote vascular endothelial growth factor- (VEGF-) mediated angiogenesis and liver regeneration. This depends on the recruitment of activated myofibroblasts or hepatic stellate cells (HSCs), which are responsible for the major production of HGF, to the periablational red zone [10]. However, liver ablation by different devices or models has various impacts on liver regeneration [12, 13]. Additionally, increasing data have determined that a platelet-derived growth factor (PDGF) is an important chemoattractant and activator for activated HSCs in the context of liver injury [14] and might be associated with liver regeneration.

To determine the change of liver function after nsPEF ablation and the influence of nsPEF ablation on liver regeneration as well as its molecular mechanisms, we investigated the change of serum AST and ALT and weight of ablated mice, followed by the assessment of liver regeneration after nsPEF ablation and the relationship between the liver regeneration and the HGF/c-Met pathway as well as PDGF.

## 2. Materials and Methods

### 2.1. Animals

C57BL6 male mice (10 weeks old) were purchased from the Shanghai Experimental Animal Center, Chinese Academy of Science. Animal studies were approved by the Animal Ethical Committee of Zhejiang University and conducted according to the National Institutes of Health Guide for the Care and Use of Laboratory Animals (NIH revised in 1996). All mice were kept on a 12-hour light-dark cycle in a pathogen-free animal facility with free access to food and water.

### 2.2. Pulse Generator and nsPEF Parameters

**Figure 1:**
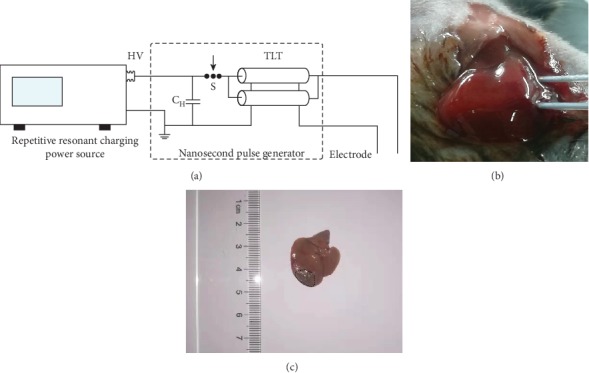
The device delivering a nanosecond pulse to the mouse liver. (a) Schematic diagram of the homemade device, including a repetitive resonant charging power source, a nanosecond pulse generator, and an electrode. (b) A representative photo of nsPEF ablation on the mouse liver after two electrodes were inserted into the liver of mice. (c) A typical photo of the ablated area on the left lobe of the mouse liver at 3 days after nsPEF ablation.

A homemade repetitive nsPEF field generator is designed (Figure 1(a)). The pulse was applied at 25 kV/cm, with a rate of 1 Hz. The pulse duration was 100 ns, and the pulse number was 300.

### 2.3. Electrode Design for Liver Ablation Experiments

The electrode was made of a 304 stainless steel needle with a diameter of 0.5 mm. Mice were generally anesthetized, and pain was relieved as previously described before operation [10]. nsPEF ablation was performed after inserting two electrodes into the left liver lobe of mice (Figure 1(b)). An oval ablated area was generated surrounding the two electrodes, which covered around 20% of total area of the left lobe (Figure 1(c)). Mice were euthanized on days 1, 3, 7, and 14 or measured for their weights at days 0, 1, 3, 5, 7, 9, 11, 13, and 15 within 14 days after nsPEF treatment.

### 2.4. Drug Administration

For inhibition of c-Met (an HGF receptor) signaling, 30 mg/kg of PHA665752 (PHA, HY-11107, MedChemExpress, USA) was injected intraperitoneally daily starting on the day of nsPEF ablation for 3 successive days (*n* = 5). For neutralizing PDGF, 2 mg/kg PDGF neutralizing antibody (ab34074, Abcam, UK) was injected into the portal vein in one day pre- and post-nsPEF ablation (*n* ≥ 3). For selective suppression of cox-2, 50 mg/kg celecoxib (SC58635, Tocris Bioscience, UK) was given daily (IP) for 3 successive days after nsPEF ablation (*n* ≥ 3). Animals were sacrificed at 3 days after nsPEF ablation.

### 2.5. Alanine Aminotransferase (ALT) and Aspartate Aminotransferase (AST) Measurement

Blood was collected from mice, and then, serum was separated via centrifugation (3000 rpm, 10 min) at room temperature. ALT and AST were examined with an ACE Alera blood chemistry analyzer (Alfa Wassermann, 402900-1, Italy).

### 2.6. Quantification of HGF and VEGF

The amount of HGF from serum and liver tissues and the level of serum VEGF were determined using enzyme-linked immunosorbent assay (ELISA) kits (ELM-HGF-CL-1, ELM-HGF-1, and ELM-VEGF, RayBiotech, USA) according to the manufacturer's instructions. An independent experiment was performed in duplicate 3 times.

### 2.7. Histologic Examination

To observe the histologic change and immune cell infiltration in the liver after ablation, liver tissues were harvested from the ablated and unablated lobes from nsPEF-treated mice on days 1, 3, 7, and 14 postablation, followed by fixation in 4% formalin for 48 hours, paraffin embedding, and preparation of 5 mm thick slices. Hematoxylin-eosin (HE) staining was performed to assess histopathological and morphologic changes of the mouse liver mediated by nsPEF ablation. Collagen deposition was evaluated by staining the liver sections with Sirius red (Sigma-Aldrich, Rehovot, Israel) diluted in picric acid. Immunohistochemistry staining (IHC) was performed to identify cell types using the following antibodies: anti-F4/80 antibody (70076, Cell Signaling Technology, USA) for monocytes, anti-neutrophil antibody (NIMP-R14) (ab2557, Abcam, UK), and anti-*α*-SMA antibody (ab124964, Abcam, UK) for activated myofibroblasts. In addition, an anti-Ki67 antibody (ab15580, Abcam, UK) was used to define proliferated cells and assess liver regeneration status; an anti-cox2 antibody (ab15191, Abcam, UK) was used to identify the expression of cox-2 at the periablated area; an anti-MCP-1 antibody (ab8101, Abcam, UK) and anti-PDGF-B antibody (ab178409, Abcam, UK) were used to show the distribution of MCP-1 and PDGF-B at the periablated area, respectively. Quantification was performed by counting the number of positive cells within 5 random fields of high-power (×100) microscopy.

### 2.8. Quantitative Real-Time PCR Analysis

Total RNA was extracted using TRIzol reagent (Sangon, B610409, China) and reversely transcribed into cDNA using HiScript II Q Select RT SuperMix for qPCR (Vazyme, R232-01, China) according to the manufacturer's instructions. Quantitative real-time PCR was carried out for detecting mRNA level of HGF with ChamQ Universal SYBR qPCR Master Mix (Vazyme, Q711-02, China). Beta-actin was used as the internal control. The primers of HGF were as follows: HGF forward primer 5′-ATGTGGGGGACCAAACTTCTG-3′ and reverse primer 5′-GGATGGCGACATGAAGCAG-3′.

### 2.9. Statistical Analysis

GraphPad Prism 5 software (La Jolla, CA) was used for statistical analysis. All data were presented as mean ± standard deviation. The statistical difference between two groups was determined by two-tailed Student's *t*-test. *P* < 0.05 was considered to have statistically significant difference.

## 3. Results

### 3.1. nsPEF Is Safe for Liver Ablation

**Figure 2:**
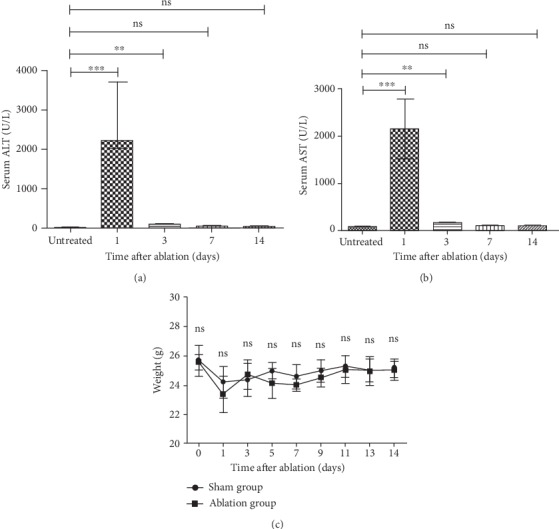
nsPEF ablation is a safe approach. (a) Serum ALT levels were measured for mice treated with nsPEF. (b) Serum AST levels were measured for mice treated with nsPEF. (c) Mouse weight was measured between 1 and 14 days after ablation. Data were presented as mean ± SD (^∗^*P* < 0.05, ^∗∗^*P* < 0.01, and ^∗∗∗^*P* < 0.001).

To investigate the safety of liver ablation by nsPEF, serum levels of ALT and AST were monitored regularly in 2 weeks after ablation. Serum levels of ALT and AST rapidly increased to a peak concentration in 24 hours, respectively, for nsPEF-treated mice (Figures 2(a) and 2(b)). However, both ALT and AST amounts could be declined to the baseline level at 7 days after ablation (Figures 2(a) and 2(b)). In addition, no significant difference in body weight was observed between mice treated with nsPEF ablation and untreated mice within 14 days (Figure 2(c)).

### 3.2. nsPEF Ablation Stimulates Robust Regeneration of the Whole Liver

**Figure 3:**
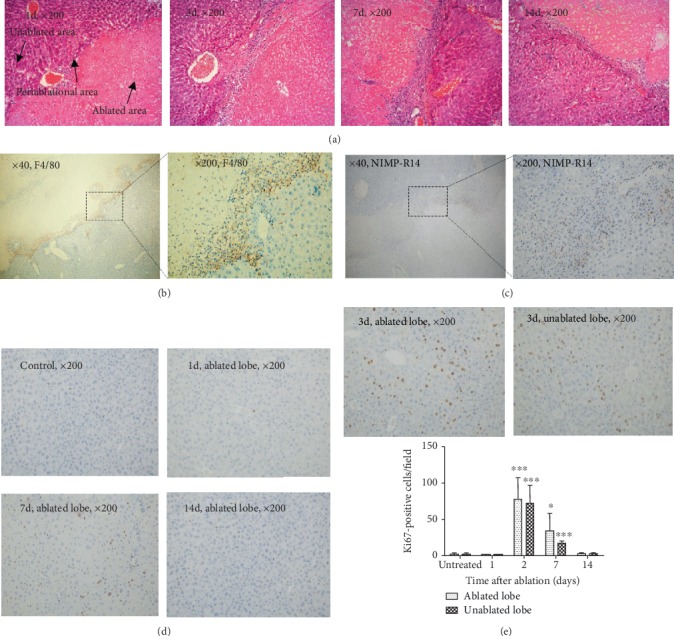
nsPEF promotes robust liver regeneration. (a) Representative images of HE staining (magnification, ×200) at different time points after liver ablation by nsPEF. (b) IHC staining for F4/80 shows macrophages accumulating at the border zone at 3 days after nsPEF ablation (original magnification, ×40 and ×200, respectively). (c) IHC staining for NIMP-R14 shows neutrophils accumulating at the border zone at 24 hours after nsPEF ablation (original magnification, ×40 and ×200, respectively). (d) Hepatic cell proliferation was detected by IHC staining for Ki67-positive staining cells within the ablated and unablated lobes of the mouse liver at indicated time points after nsPEF ablation (magnification, ×200). (e) Quantification of Ki67-positive cells in the unablated area of ablated lobes and unablated lobes within 14 days in indicated time points after nsPEF ablation. The number of positive cells was counted by the average number of positive cells in 5 random fields of a ×100 microscope. Data were presented as mean ± SD (^∗^*P* < 0.05, ^∗∗^*P* < 0.01, and ^∗∗∗^*P* < 0.001).

Given that RFA potentially promotes liver growth [10], IHC staining for Ki67, a typical marker for cell proliferation, was employed to determine the effect of nsPEF ablation on liver regeneration. Liver tissues after ablation by nsPEF were characterized by a clear boundary surrounding ablation zone, which divided the ablated lobe into three parts: ablated area, unablated area, and periablational area (Figure 3(a)) with accumulation of neutrophils (Figure 3(b)) and macrophages (Figure 3(c)) after ablation. As expected, Ki67 exhibited significantly high expression within hepatic cells in the unablated area of ablated lobes (the lobes with nsPEF ablation) as well as unablated lobes (the lobes without nsPEF ablation) from 3 to 7 days after ablation, and its peak amount appeared in 3 days after ablation (Figures 3(d) and 3(e)). However, no significant difference of the number of Ki67-positive cells within liver tissues was noticed between ablated mice and untreated mice at day 14 (Figures 3(d) and 3(e)).

### 3.3. nsPEF-Mediated Liver Regeneration Relies on the Activation of the HGF/c-Met Pathway

**Figure 4:**
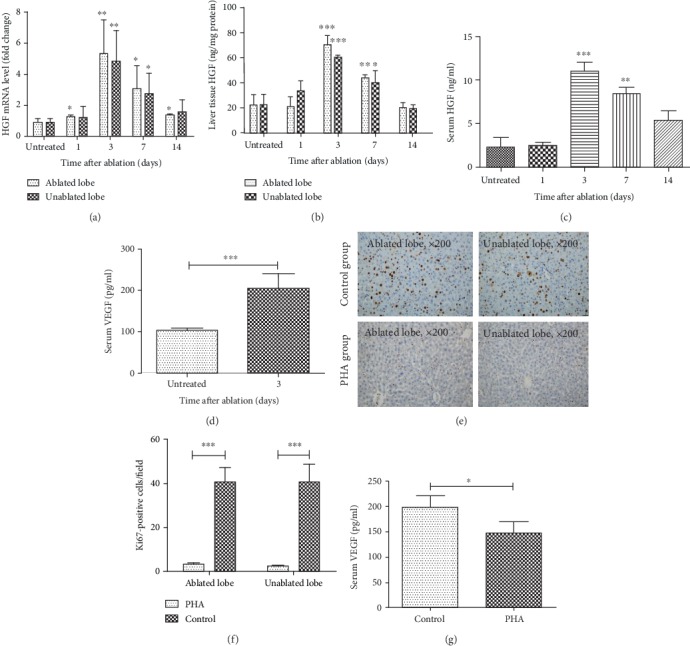
The liver regeneration induced by nsPEF is related to the activated HGF/c-Met pathway. (a, b) Level of HGF mRNA (a) and protein (b) within liver tissues at the ablated lobe and unablated lobe at indicated time points after nsPEF ablation detected by qRT-PCR and ELISA, respectively. (c, d) Serum level of HGF (c) and VEGF (d) examined by ELISA at indicated time points after nsPEF ablation. (e–g) Mice were administered intraperitoneally with the c-Met inhibitor PHA (30 mg/kg) (PHA) or vehicle (control) daily for 3 successive days after nsPEF ablation. Then, the liver and serum were harvested for the detection of Ki67-positive hepatocytes by IHC staining and serum VEGF level by ELISA. Representative IHC staining results with magnification of ×200 (e), the quantification of the average number of Ki67-positive cells within 5 random fields of ×100 microscopy (f), and serum VEGF level (g) by ELISA on day 3 after ablation. Data were presented as mean ± SD (^∗^*P* < 0.05, ^∗∗^*P* < 0.01, and ^∗∗∗^*P* < 0.001).

Taking into consideration the significant role of HGF/c-Met signaling in liver regeneration, we assessed the level of several cytokines related to the HGF/c-Met pathway in the serum or liver tissues of ablated mice by qRT-PCR and ELISA to clarify the underlying mechanism of liver regeneration after nsPEF ablation. The results demonstrated that mRNA level of HGF was significantly increased within liver tissues of nsPEF-treated mice, and its highest levels within ablated and unablated lobes were 5.9 and 5.3 times more than those of counterparts from untreated mice, respectively, at 3 days after ablation (Figure 4(a)). In agreement, the protein level of HGF was dramatically elevated within liver tissue and climbed to the peak in 3 days after ablation (Figure 4(b)). Similarly, the serum level of HGF protein also showed significant increases after ablation with a peak level at 3 days (Figure 4(c)). In addition, the amount of VEGF in serum, a key downstream cytokine of c-Met, also increased strikingly, with 2 times higher than that in untreated mice in 3 days after nsPEF ablation (Figure 4(d)).

In order to further confirm the key regulation of HGF/c-Met signaling on liver regeneration, the c-Met inhibitor PHA665752 (PHA) was administered after liver nsPEF ablation on mice. With expectancy, the number of the Ki67-positive staining cells was significantly decreased at 3 days after nsPEF ablation (Figures 4(e) and 4(f)). Moreover, the serum level of VEGF was also decreased markedly in the PHA group in comparison to the control group at the same time point (Figure 4(g)).

### 3.4. PDGF Upregulation Is Responsible for nsPEF-Induced High Expression of HGF and Liver Regeneration

**Figure 5:**
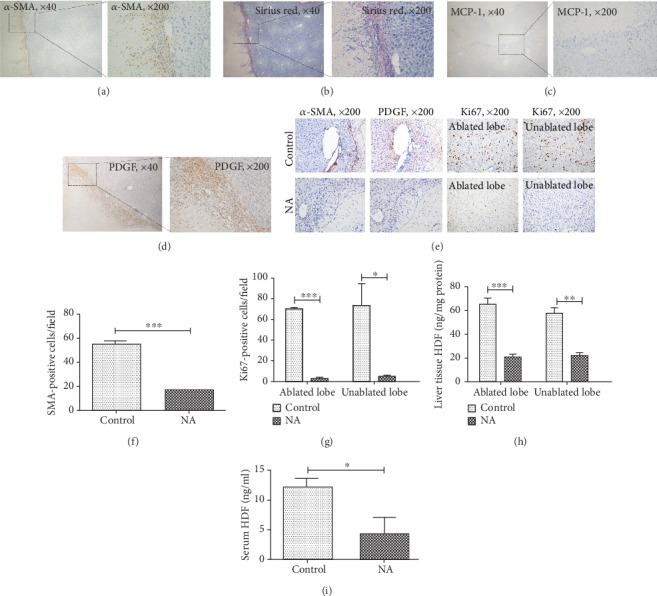
Upregulation of PDGF at the periablational area induced liver regeneration after nsPEF ablation through recruitment of activated HSCs. (a) *α*-Smooth muscle actin (*α*-SMA) staining (original magnification, ×40 and ×200) shows the distribution of activated myofibroblasts (activated HSCs) at the border zone at 3 days after nsPEF ablation. (b) Sirius red staining (original magnification, ×200) shows collagen deposition at the border zone at 3 days after nsPEF ablation. (c, d) Representative images of IHC staining for two key molecules for recruitment and activation of HSCs, MCP-1 and PDGF within the periablational area in 3 days after ablation (original magnification, ×40 and ×200). (e–i) A PDGF neutralization IgG antibody (NA) or an isotype IgG antibody (control) were injected into the portal vein of mice in one day pre- and postablation, respectively. Three days after nsPEF ablation, liver tissues from both ablated and unablated lobes were harvested for examining PDGF expression by IHC staining for PDGF, activated HSCs by IHC staining for *α*-SMA, and liver regeneration by IHC staining for Ki67. Representative images of IHC staining for PDGF, *α*-SMA, and Ki67 (magnification, ×200) (e) and quantification of *α*-SMA- (f) or Ki67- (g) positive staining cells within 5 random fields of ×100 microscopy. The amount of HGF was detected within liver tissues (h) and serum (i) by ELISA. Data were presented as mean ± SD (^∗^*P* < 0.05, ^∗∗^*P* < 0.01, and ^∗∗∗^*P* < 0.001).

Previous studies have shown that HGF is mainly produced by activated myofibroblasts or HSCs once liver injury occurs [15], and monocyte chemotactic protein 1 (MCP-1) and PDGF are the two most potent chemoattractants for recruitment of activated HSCs rather than quiescent HSCs [14]. Meanwhile, the accumulation of activated HSCs is featured as the collagen deposition [14], which was confirmed by the positive IHC staining for *α*-SMA and Sirius red staining within the periablational zone at 3 days after nsPEF ablation (Figures 5(a) and 5(b)). Thus, the expression levels of MCP-1 and PDGF were examined. According to IHC results, PDGF but not MCP-1 was highly expressed within the periablational zone (Figures 5(c) and 5(d)). Furthermore, the treatment with a PDGF-specific neutralizing antibody was able to inhibit accumulation of activated HSCs, characterized by the decreased number of *α*-SMA-positive staining cells within the periablational area (Figures 5(e) and 5(f)).

Of interest, liver regeneration was also hampered markedly by NA, indicated as the declined number of Ki67-positive staining cells (Figures 5(e) and 5(g)). In addition, HGF levels both within liver tissues and serum in the NA group were much lower than those in the control group at 3 days after ablation (Figures 5(h) and 5(i)).

### 3.5. nsPEF-Mediated Liver Regeneration Is More Obvious within the Periablational Area Dependent on HGF/c-Met Activation

**Figure 6:**
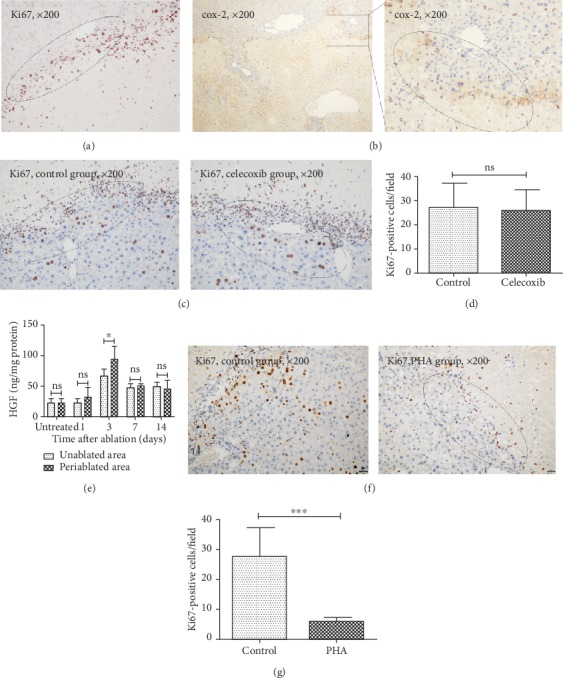
nsPEF induces a more obvious hepatocyte proliferation at the periablational area in comparison to other unablated areas at ablated lobes. (a) Representative image of IHC staining for Ki67 (original magnification, ×200) shows more obvious hepatocyte proliferation at the periablational zone at 3 days after nsPEF ablation compared to other areas at the ablated lobe. (b) Representative image of IHC staining for cox-2 (original magnification, ×40 and ×200) at the periablational zone at 24 hours after nsPEF ablation. (c, d) Mice were administered intraperitoneally with the cox-2 inhibitor celecoxib (50 mg/kg) or vehicle (control) daily for 3 successive days after nsPEF ablation. Then, livers were harvested for the detection of Ki67-positive staining cells. Representative results of IHC staining with magnification of ×200 at the periablational area of the ablated lobe of mice from the celecoxib group and control group (c) and quantification of Ki67-positive cells within 5 random fields of ×100 microscopy (d). (e) Level of HGF within liver tissues in the unablated area and periablated area within the ablated lobe at indicated time points after nsPEF ablation detected by ELISA, respectively. (f, g) Mice were administered intraperitoneally with the c-Met inhibitor PHA (30 mg/kg) (PHA) or vehicle (control) daily for 3 successive days after nsPEF ablation. Then, livers were harvested for the detection of Ki67-positive staining hepatocytes. Representative results of IHC staining for Ki67 with magnification of ×200 at the periablational area of the ablated lobe of mice from the PHA group and control group (f) and quantification of Ki67-positive cells within 5 random fields of ×100 microscopy (g). Data were presented as mean ± SD (^∗^*P* < 0.05, ^∗∗^*P* < 0.01, and ^∗∗∗^*P* < 0.001).

As shown in Figure 6(a), more Ki67-positive staining cells were noticed within the periablational area than within other areas, mirroring that cells within this area had the higher proliferation. Because increased cox-2 expression in the periablational area after hepatic RFA has been reported to induce higher proliferation of hepatocytes [16], we tested cox-2 expression in the periablational area in ablated lobes at 24 hours after nsPEF ablation. However, the result showed that low level of cox-2 expression was noticed (Figure 6(b)) and the higher cell proliferation here at 3 days after ablation could not be suppressed by celecoxib, a cox-2 inhibitor (Figures 6(c) and 6(d)). Intriguingly, higher level of HGF within the periablational area was observed in comparison to other unablated areas (Figure 6(e)), and the higher proliferation of cells within the periablational area could be significantly inhibited after administration of PHA (Figures 6(f) and 6(g)), suggesting that higher cell proliferation within the periablational area was still dependent on HGF/c-Met activation.

## 4. Discussion

Recently, global liver regeneration stimulated by RFA has gained great attentions, and this phenomenon is likely due to the accumulation of stromal cells, including inflammatory cells and HSCs, within the boundary area around the ablation zone [10]. Here, a similar result was observed in our model of nsPEF-treated mice, and liver regeneration occurred within both ablated and unablated lobes from mice receiving liver nsPEF ablation. In particular, the proliferation of cells was more obvious within the periablational area which was featured by the accumulation of activated HSCs.

Unfortunately, RFA has been reported to lead to great and lasting damage to liver function [17], and this side effect is a huge challenge and cannot be neglected when RFA is applied to treat liver lesions. As an analogue to RFA, nsPEF ablation on the liver also had an impact on liver function in the first 3 days after ablation. Nonetheless, the impaired liver function was able to recover to normal level at 7 days after nsPEF ablation, suggesting that the damage to liver function induced by nsPEF was transient and reversible due to little thermal injures it caused, similar to the findings by Nuccitelli et al. [18]. Moreover, nsPEF ablation merely affected weight of mice on the account of no difference of weight between nsPEF-treated and untreated mice. These observations provide a clue to the safety of application of nsPEF ablation on the liver.

HGF is a well-known “complete mitogen” that binds to tyrosine receptor kinase c-Met resulting in signaling transduction and ultimately DNA synthesis within hepatocytes and the proliferation of hepatocytes [19]. HGF production mainly relies on the activation of HSCs after the damage to the liver [15]. Our results confirmed the accumulation of activated HSCs and collagen deposition at the periablational area after nsPEF ablation. In parallel, HGF levels in both serum and liver tissues at all lobes were elevated from 3 to 14 days after nsPEFs albeit a downward trend during this period. Interestingly, the result of Ki67 staining showed the cell proliferation peak (at both unablated areas in the ablated lobe and unablated lobes) appeared at 3 days after ablation, different from that at 7 days in the RFA model, which could be attributed to the earlier peak of serum HGF level in the nsPEF ablation model [10]. In addition, blockade of the HGF/c-Met pathway led to a significant suppression of cell proliferation and liver regeneration. Thus, the accumulation of activated HSCs was involved in liver regeneration after nsPEF ablation, which induced activation of the HGF/c-Met pathway [10].

The recruitment or activation of HSCs has been well described and relies on PDGF expression [14, 20]. In our model, nsPEF ablation caused an injured area in the liver, which activated Kupffer cells to release high level of PDGF at the periablational area [21, 22]. Besides, liver injury was associated with increased autocrine PDGF for HSCs, and this positive feed-back loop aids in enlarging local accumulation and activation of HSCs [20]. Our data determined that PDGF was highly expressed within the periablational area where there was a noticed accumulation of HSCs after nsPEF ablation. Furthermore, after the use of the PDGF neutralizing antibody, the accumulation of activated HSCs within the periablational area, the liver regeneration, and the increased level of HGF in serum and liver tissues were strikingly suppressed after nsPEF ablation. These data showed that the liver regeneration induced by the rise of HGF after nsPEF ablation was reliable on PDGF.

This study also demonstrated that liver regeneration was more obvious within the periablational area than within any other areas. These phenomena might be addressed by the idea that higher expression of HGF would bring extra benefit to liver regeneration here. However, different from the significant impact of increased expression of cox-2 on higher proliferation of hepatocytes at the periablational area in the hepatic RFA model, cox-2 might not function as an important factor in similar higher cell proliferation at the periablational area in our hepatic nsPEF ablation model due to much lower expression of cox-2 here. Additionally, several other limitations in the current study still need to be presented. Firstly, the model of nsPEF treatment was used on the basis of only one set of parameters. Secondly, the impact of nsPEF ablation on progression or recurrence of liver cancer was not evaluated because one of the most important applications of nsPEF ablation was to treat HCC.

In conclusion, our findings showed that nsPEF ablation was safely applied on the liver and stimulated global liver regeneration dependent on the PDGF/HGF/c-Met pathway. Our work provides preclinical evidences that assess the safety and potential effects of nsPEF application on treating liver lesions; the PDGF/HGF/c-Met pathway would be a potential target for regulating liver regeneration after nsPEF ablation.

## Data Availability

The data used to support the findings of this study are included within the article.
